# Supplementary figures and images for: Bamgineer: Introduction of simulated allele-specific copy number variants into exome and targeted sequence data sets
Source: PLoS Comput Biol. 2018 Mar 28;14(3):e1006080. doi: 10.1371/journal.pcbi.1006080 (PMC5891060; doi:10.1371/journal.pcbi.1006080)

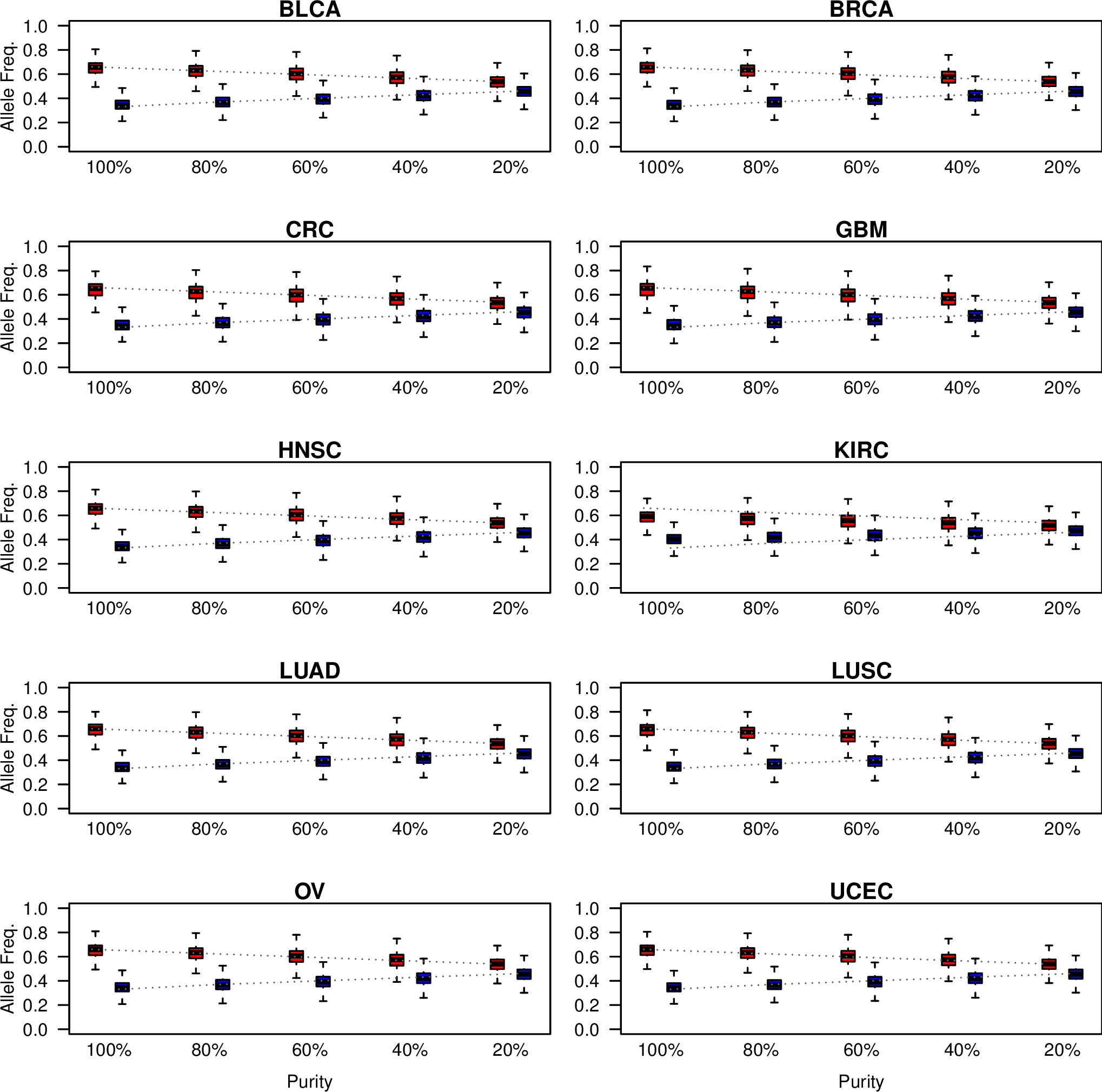

Supplement: S1 Fig — Allelic ratio boxplots for cancer-specific copy number gain at heterozygous SNP loci for haplotypes affected (blue) and Haplotypes not affected (red) at different tumour cellularity levels. (TIF) [file pcbi.1006080.s001.tif]

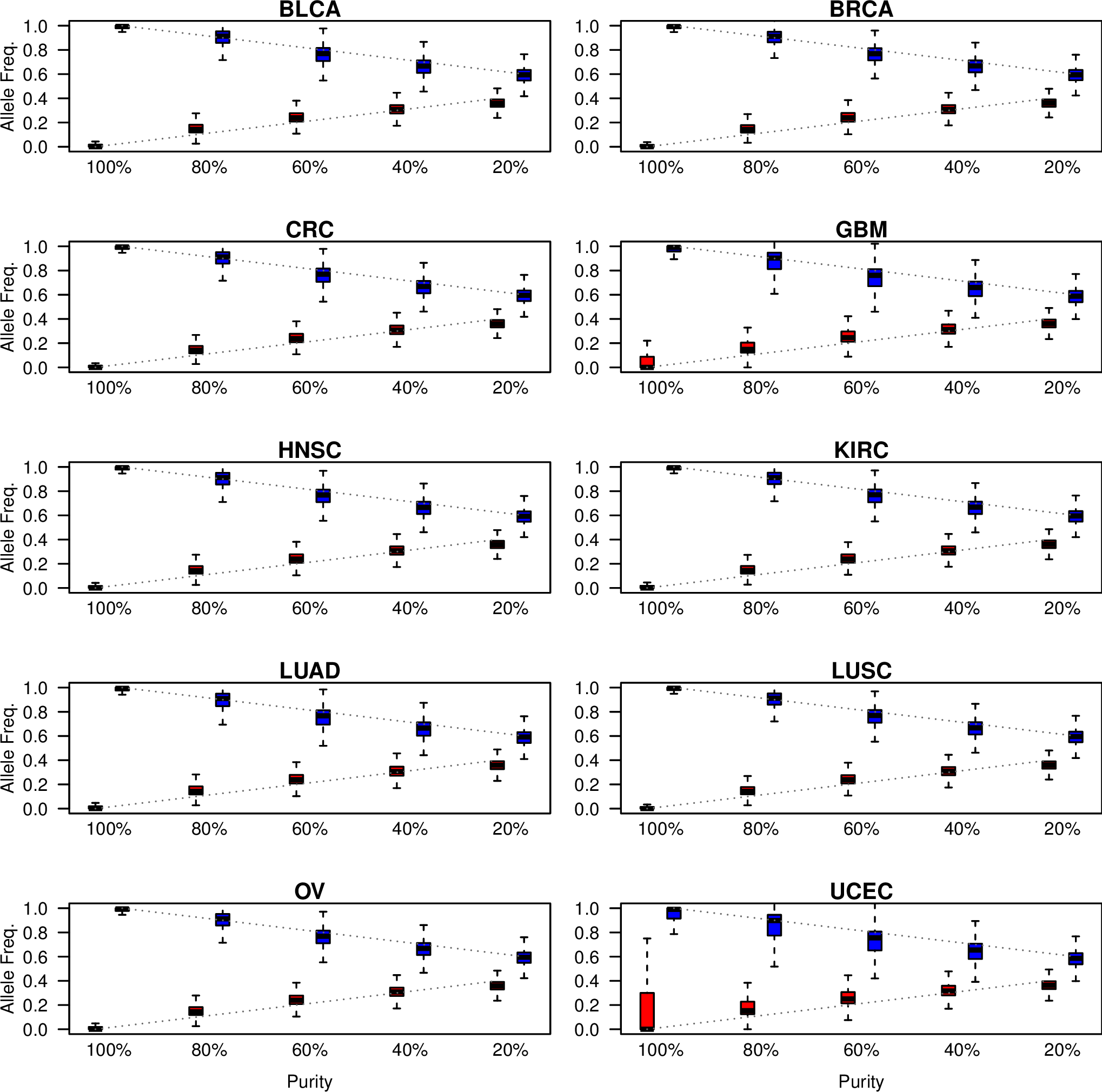

Supplement: S2 Fig — Allelic ratio boxplots for cancer-specific copy number loss at heterozygous SNP loci for haplotypes affected (blue) and Haplotypes not affected (red) at different tumour cellularity levels. (TIF) [file pcbi.1006080.s002.tif]

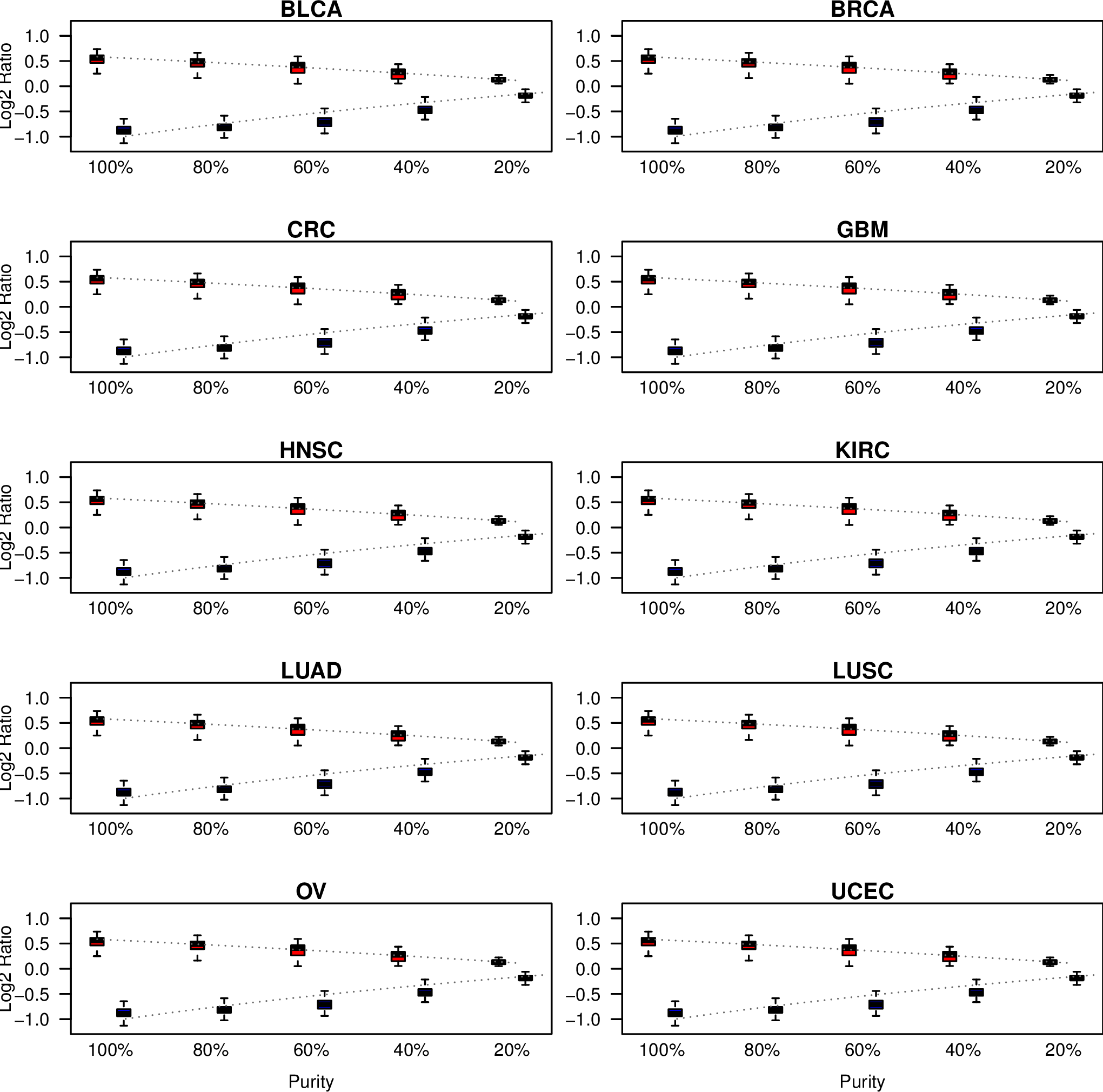

Supplement: S3 Fig — Tumor to normal log2 depth ratio boxplots for cancer-specific copy number gain (red) and loss (blue) at different tumour cellularity levels normalized for mean ploidy. (TIF) [file pcbi.1006080.s003.tif]

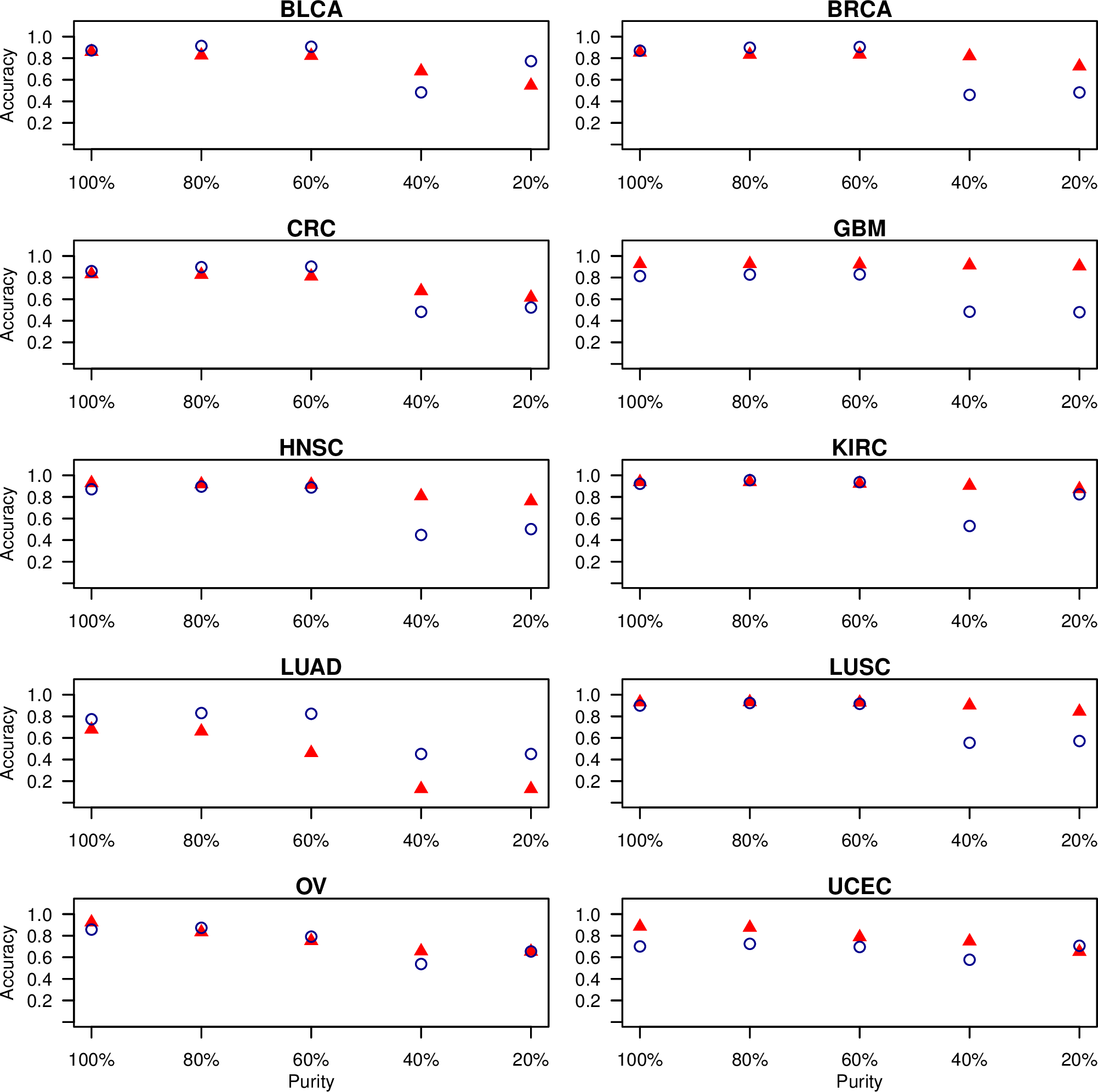

Supplement: S4 Fig — (TIF) [file pcbi.1006080.s004.tif]

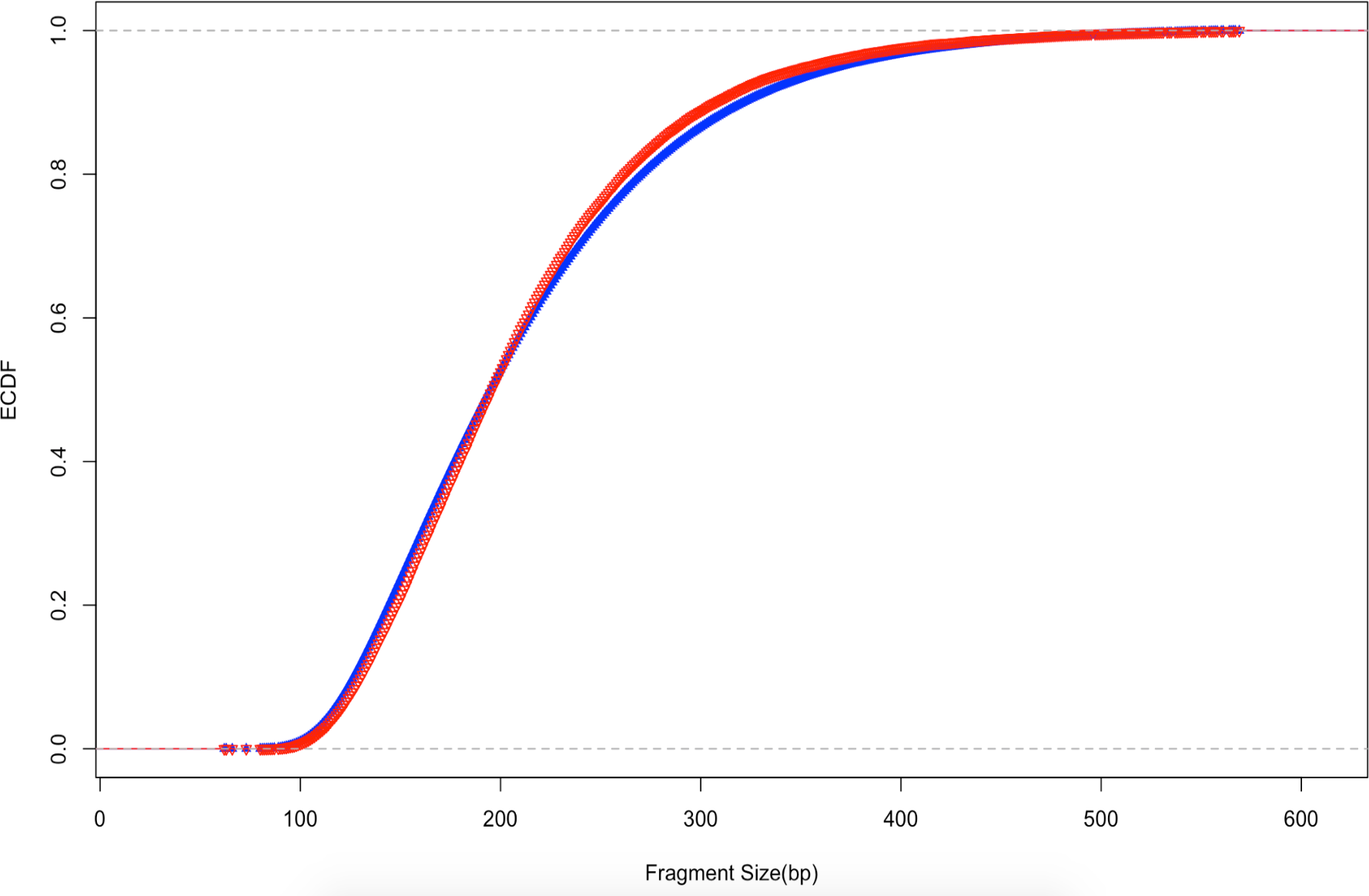

Supplement: S5 Fig — Experimental Cumulative Density Functions (ECDF) of all fragment lengths (blue; median 194, mean: 212.3) and newly introduced read pairs (red; median 194, mean: 211.97) allele specific gain for chromosome 22. The distribution before and after the addition of CNV is consistent. (TIF) [file pcbi.1006080.s005.tif]

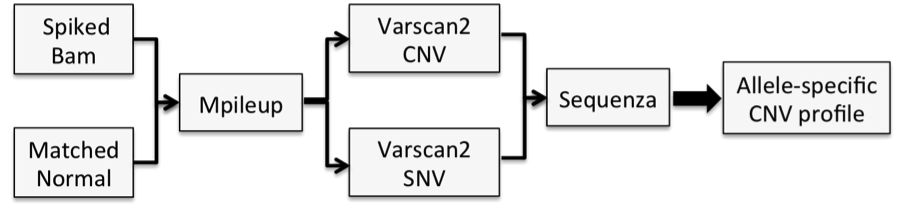

Supplement: S6 Fig — Pipeline for detecting absolute and allele-specific CNV. Varscan2 was used for depth normalization. Varscan2 output was then used to infer allele-specific copy number profiles as well as tumour purity and ploidy using Sequenza note that in this case “tumour” file will be the synthetic file generated using Bamgineer. (TIF) [file pcbi.1006080.s006.tif]

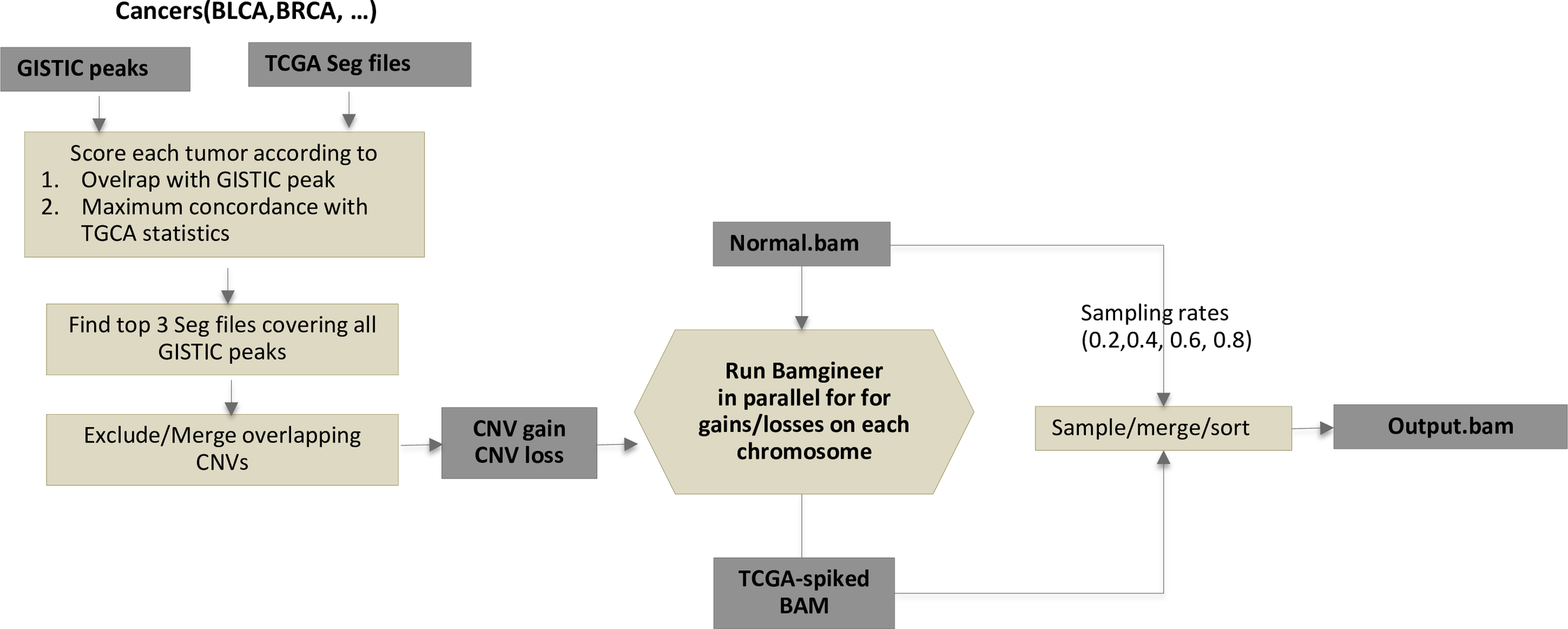

Supplement: S7 Fig — Overview of the design used to introduce cancer-specific CNV events. Parallelization module enables to simultaneously implement cancer-based, chromosome-based and event-based engineering of CNVs, significantly improving the performance (see “Runtime benchmarks and parallelization”) (TIF) [file pcbi.1006080.s007.tif]

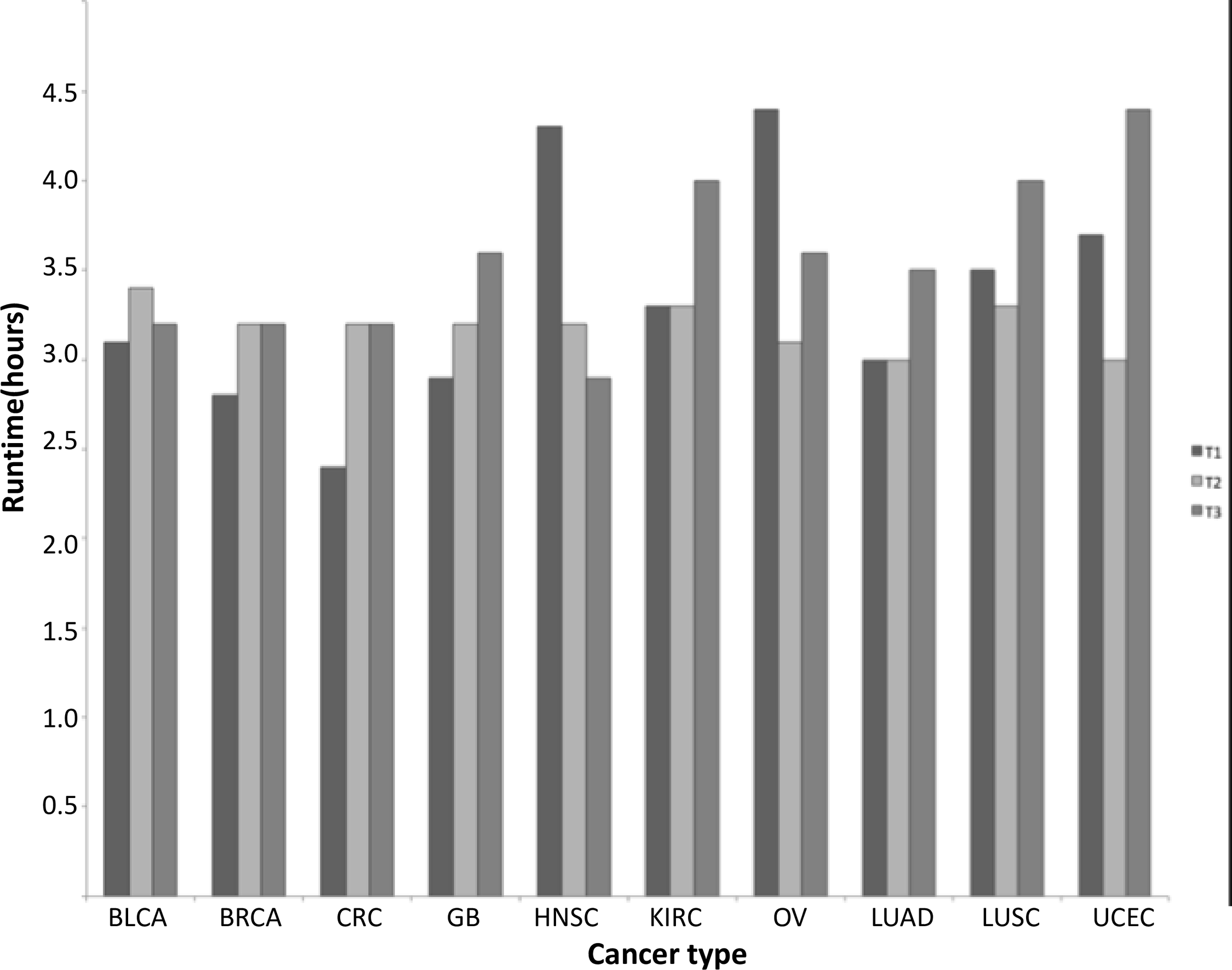

Supplement: S8 Fig — T1, T2, and T3 represent the three exemplar tumors selected from TCGA tumor profiles to best represent the copy number landscape for each cancer type for each cancer type (see Table 1). (TIF) [file pcbi.1006080.s008.tif]

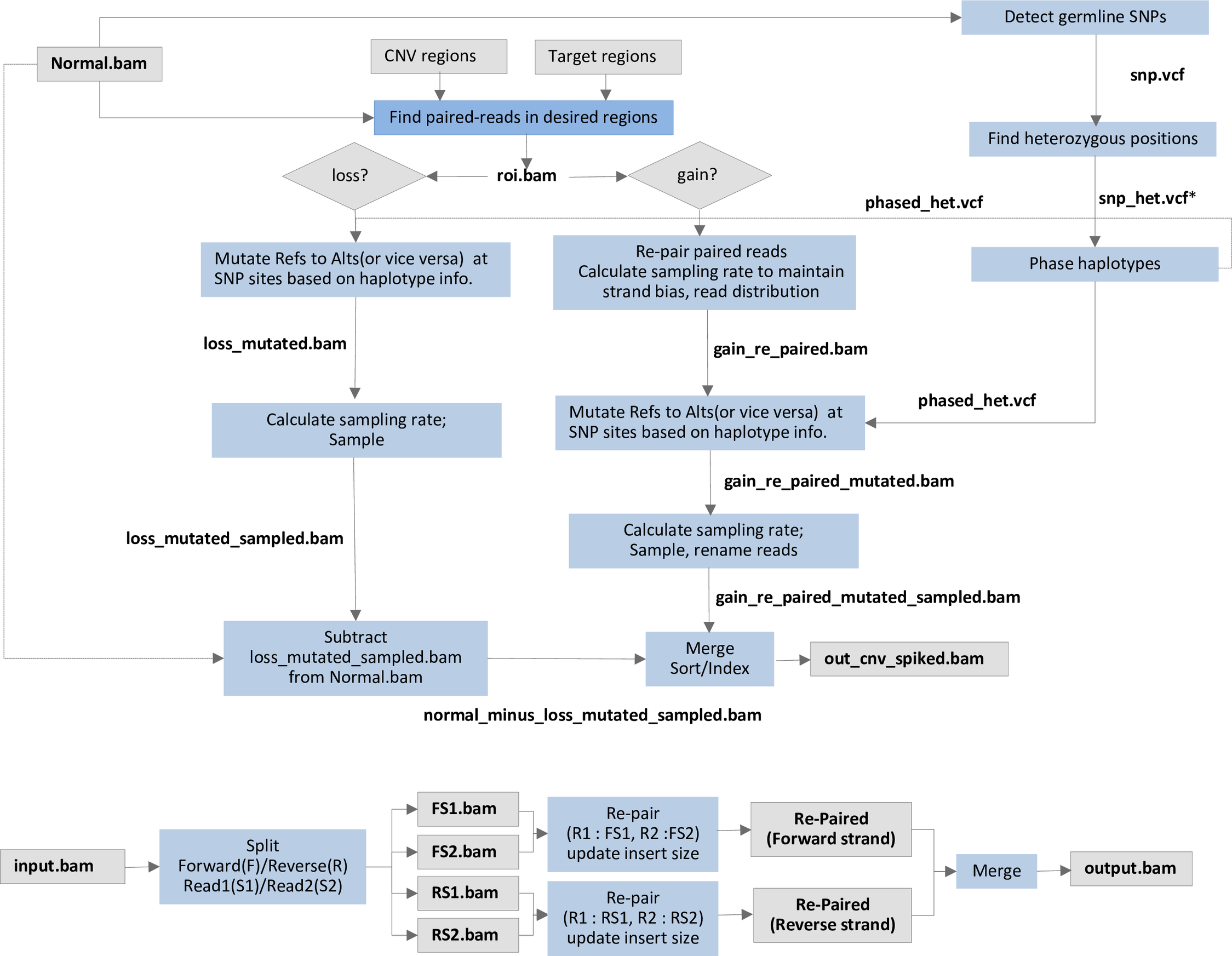

Supplement: S9 Fig — A) Overall architecture of Bamgineer for editing an existing BAM file to add and delete the user defined CNV event. The input and output files are shown in dark grey. The modules are shown darker relative to the files generated at each step. B) Creating new paired-reads from existing ones. The algorithms splits the input BAM files into four separate files according to DNA strand and read information. Bamgineer then iterates through split reads (read1 and read2) from each strand separately, pairing one read from read1 splits to another read from read2 split. The insert-size (tlen) in the newly paired read is then calculated and updated. (TIF) [file pcbi.1006080.s009.tif]

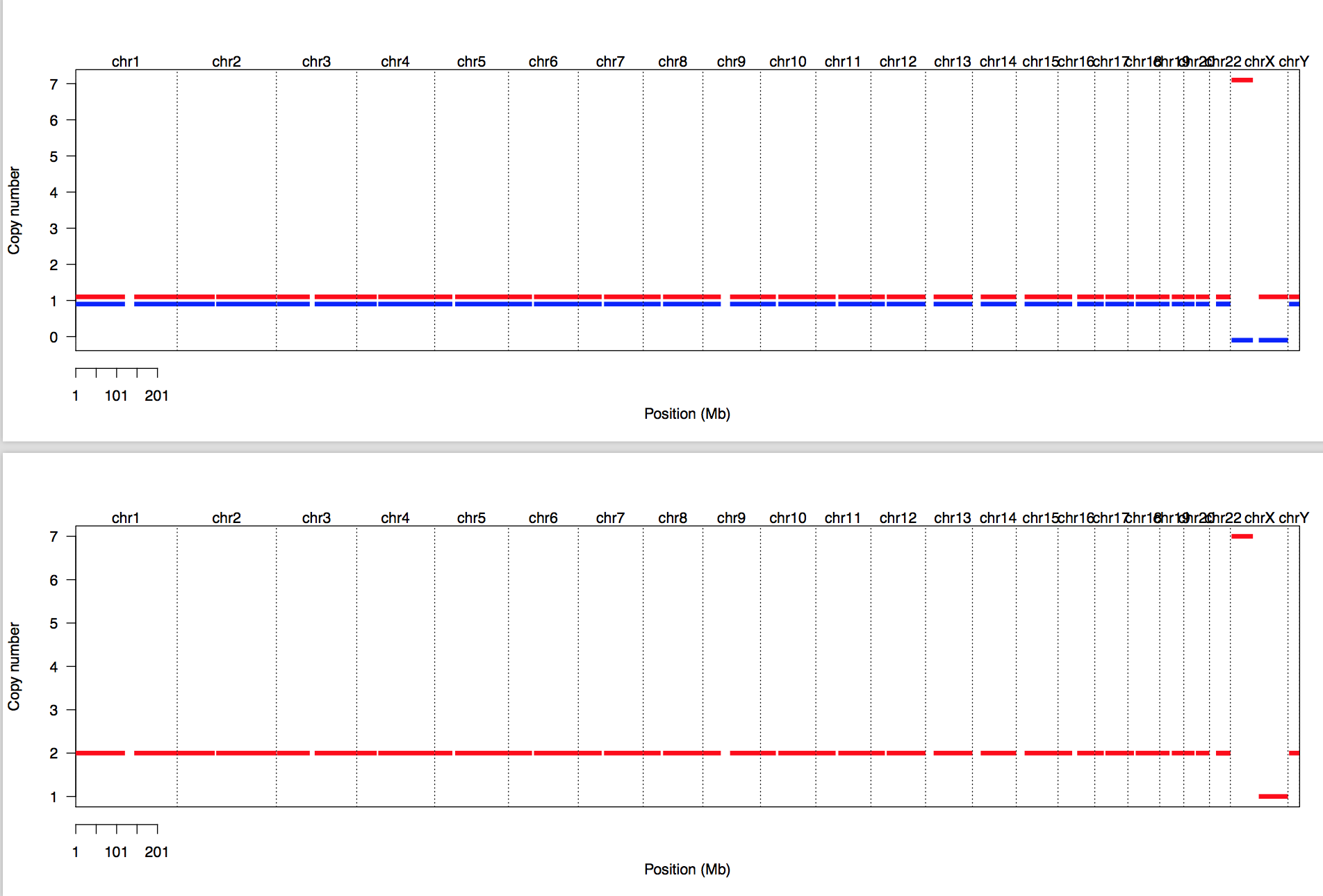

Supplement: S10 Fig — Bamgineer was used to introduce of 6 additional copies of the p-arm of chromosome X to total copy number of 7 from whole exome sequencing data from a male and accurately called using Sequenza. In the top track, blue and red lines show allele specific copy number profiles for each chromosome (lines are offset from discrete copy number values by ± 0.1 for visual separation of the two alleles). In the bottom track, the red lines depict total copy number with an overall normal diploid genome apart from the amplification of Xp introduced by Bamgineer. (TIF) [file pcbi.1006080.s010.tif]
