# Supplementary material for: Bamgineer: Introduction of simulated allele-specific copy number variants into exome and targeted sequence data sets
Source: PLoS Comput Biol. 2018 Mar 28;14(3):e1006080. doi: 10.1371/journal.pcbi.1006080 (PMC5891060; doi:10.1371/journal.pcbi.1006080)
Supplement: S1 Table — Introduction of EGFR gain to targeted 5-gene panel (18 kb) applied to a cell-free DNA at frequencies of 100, 10, 1, 0.1, and 0.01%. The Alt and Ref columns represent the count of alternative and reference base pairs at each variant position. The columns in bold represents the phased targeted haplotype. We note that Bamgineer can be used to introduce subtle shifts in coverage of specific allelic variants, and haplotype representation consistent with the targeted allele frequencies. (DOCX) [file pcbi.1006080.s011.docx]

**S1 Table. Allele counts at 3 SNPS** **in *EGFR.*** Introduction of *EGFR* gain to targeted 5-gene panel (18 kb) applied to a cell-free DNA at frequencies of 100, 10, 1, 0.1, and 0.01%. The Alt and Ref columns represent the count of alternative and reference base pairs at each variant position. The columns in bold represents the phased targeted haplotype. We note that Bamgineer can be used to introduce subtle shifts in coverage of specific allelic variants, and haplotype representation consistent with the targeted allele frequencies.

| Tumour Purity | Alt | Ref | | Exp.(Alt) | | Exp.(Ref) | | |
| --- | --- | --- | --- | --- | --- | --- | --- | --- |
| SNP1 (rs2227983) | | | | | | | |  |
| Original BAM | 32260 | | **31769** | | 32260 | | **31769** |  |
| 100% | 32260 | | **63542** | | 32260 | | **63538** |  |
| 10% | 32260 | | **34944** | | 32260 | | **34946** |  |
| 1% | 32260 | | **32089** | | 32260 | | **32086** |  |
| 0.10% | 32260 | | **31803** | | 32260 | | **31800** |  |
| 0.01% | 32260 | | **31774** | | 32260 | | **31772** |  |
| SNP2 (rs10228436) | | | | | | | |  |
| Original BAM | **36806** | | 34971 | | **36806** | | 34971 |  |
| 100% | **73614** | | 34971 | | **73612** | | 34971 |  |
| 10% | **40531** | | 34971 | | **40486** | | 34971 |  |
| 1% | **37182** | | 34971 | | **37174** | | 34971 |  |
| 0.10% | **36843** | | 34971 | | **36842** | | 34971 |  |
| 0.01% | **36810** | | 34971 | | **36809** | | 34971 |  |
| SNP3 (rs17337023) | | | | | | | |  |
| Original BAM | **29527** | | 28865 | | **29527** | | 28865 |  |
| 100% | **59057** | | 28865 | | **59054** | | 28865 |  |
| 10% | **32413** | | 28865 | | **32479** | | 28865 |  |
| 1% | **29834** | | 28865 | | **29822** | | 28865 |  |
| 0.10% | **29560** | | 28865 | | **29556** | | 28865 |  |
| 0.01% | **29530** | | 28865 | | **29529** | | 28865 |  |
